# The Metabolic Regulation of the NKG2D-Positive NK and T Cells and Their Role in Disease Progression

**DOI:** 10.3390/biom15111506

**Published:** 2025-10-24

**Authors:** Jiayi Tang, Yaqi Lu, Min Chen, Qifan Wu, Yifei Li, Yingqiao Qin, Shaomei Liang, Sulan Luo, Kunpeng Liu

**Affiliations:** Guangxi Key Laboratory of Special Biomedicine, School of Medicine, Guangxi University, Nanning 530004, China; 2328391039@st.gxu.edu.cn (J.T.); 2428391028@st.gxu.edu.cn (Y.L.); 2328391003@st.gxu.edu.cn (M.C.); 2328391047@st.gxu.edu.cn (Q.W.); 2328302009@st.gxu.edu.cn (Y.L.); 2237030141@st.gxu.edu.cn (Y.Q.); 2237030137@st.gxu.edu.cn (S.L.)

**Keywords:** NKG2D, natural killer cells, metabolic regulation, metabolic disease, cell therapy

## Abstract

Natural killer (NK) cells are the main cytotoxic lymphocytes of the natural immune system, which play an important role in tumor immune surveillance and anti-viral response. The surface receptor NKG2D can recognize NKG2D ligands on the surface of tumor or metabolism-stressed cells, thereby activating immune responses and mediating cytotoxicity and anti-tumor activity of NK cells. However, NKG2D-positive NK cells are regulated by metabolites, and play a negative role in metabolic diseases. Various metabolites, including lipids, reactive oxygen species (ROS), glucose and amino acids, regulate NKG2D expression and NK cell activity and decide the immune microenvironment of pathological tissue. Thus, targeted therapies based on NKG2D-positive NK cell have entirely different strategies in the treatment of tumor or metabolic diseases. This article focuses on the metabolic regulation of NKG2D-positive NK cells and their opposite roles in disease progression, including of cancer and metabolic disease. In the future, in-depth studies of the regulatory mechanisms of the NKG2D signaling pathway by metabolites and the optimization of the safety and efficacy of targeted therapeutic strategies will lead to new breakthroughs in the treatment of tumors and metabolic diseases, providing patients with more effective treatment options.

## 1. Introduction

NK cells are the main cytotoxic lymphocytes of the natural immune system, which play an important role in tumor immune surveillance and antiviral response. NK cells originate from bone marrow hematopoietic stem cells, making up 5–15% of lymphocytes in peripheral blood. Their surface receptors are classified into two categories: killer cell immunoglobulin-like receptors (KIR) and natural cytotoxicity receptors (NCR). These receptors determine whether to initiate a killing program by recognizing either Major Histocompatibility Complex (MHC) class I molecules or stress-induced proteins on target cells. When the expression of MHC-I molecules on target cells is downregulated (a common occurrence in tumor or virus-infected cells), the inhibitory signals in NK cells are diminished and activating signals predominate, thereby triggering the release of cytotoxic granules such as perforin and granzymes.

The activation of NK cells is regulated by the balance between their activating and inhibitory receptors [1], a finely tuned mechanism that enables NK cells to effectively combat tumors and viral infections. NK cells recognize tumor cells or stressed and infected cells through the activating receptor NKG2D, which binds to NKG2D ligands expressed on the surface of these cells. This interaction activates the immune response and mediates the killing of these cells, thereby contributing to tumor immune surveillance and clearance. In addition to their direct cytotoxic actions, NK cells secrete cytokines such as interferon-γ (IFN-γ) and tumor necrosis factor-α (TNF-α), which stimulate other immune cells, such as dendritic cells (DCs) [2], thereby participating in adaptive immune responses. This dual functionality allows NK cells to play a central role in the immune system, as they are able to rapidly respond to pathogens and tumor cells while also enhancing the overall immune response by modulating the functions of other immune cells.

NK cell activity is regulated by metabolism. Studies have shown that glycolysis plays an important role in modulating NK cell activity. To enhance their cytotoxicity and cytokines secretion, NK cells regulate both glycolysis and oxidative phosphorylation (OXPHOS) upon activation [3,4]. Glycolysis provides a rapid means of producing energy to meet the demands of the immune response, while OXPHOS produces higher amounts of adenosine triphosphate (ATP) to maintain NK cell function. Recently, the impact of lipid metabolism on NK cell activity has also attracted increasing attention. Some studies have found that ferroptosis—a novel form of cell death precisely regulated by iron metabolism, antioxidant processes, and lipid metabolism—plays an indispensable role in the development of many diseases [5]. The maintenance of cellular metabolic homeostasis relies on a multilayered regulatory network both between the organism and its cells and within the cells themselves. The AMP-activated protein kinase (AMPK) and the mechanistic target of rapamycin (mTOR) signaling pathways exert comprehensive control over both anabolic and catabolic processes. In NK cells, these pathways may influence activity by modulating metabolic processes. The availability of nutrients is critical for NK cell activity. Metabolic fuels such as glucose, amino acids, and fatty acids are essential for NK cells to perform their effector functions—including proliferation, cytotoxicity, and interferon-γ production [3]. In conditions of nutrient deprivation, NK cell activity may be suppressed.

NK cells are involved in regulating metabolism. NK cells play an important role in the development of non-alcoholic steatohepatitis (NASH) and hepatic fibrosis because they recognize NKG2D ligands on the liver cell surface via the NKG2D receptor [6]. NK cells also contribute to other pathological processes, such as atherosclerosis, by secreting inflammatory mediators to modulate immune responses [7].

In recent years, with the rapid development of immunotherapy technologies, the study of NK cells and their key signaling pathways—such as the NKG2D receptor-ligand axis—has become a hot topic in oncology and immunology. Studies have shown that enhancing the expression of either the NKG2D receptor or its ligands can improve NK cells’ ability to recognize and kill tumor cells. For instance, bi-specific antibodies can redirect effector cells expressing NKG2D to tumor cells, thereby enhancing anti-tumor activity. In addition, Chimeric Antigen Receptor (CAR)-NK cell therapy, through engineering NK cells to express a NKG2D-based CAR, can significantly improve the clearance of tumor cells [8]. These functions make NK cells versatile players in the immune system—they not only serve as the first line of defense in innate immunity but are also closely linked to the adaptive immune system in jointly maintaining the body’s health. Therefore, this article summarizes recent advances: metabolism plays a central role in regulating NK cell activity via NKG2D by influencing the expression of NKG2D ligands and reprogramming the metabolic state of NK cells, thereby altering their activity and function. NK cells also have other receptors. NKG2A is an inhibitory receptor that often pairs with CD94. Together, they are called CD94/NKG2A. This complex is found mainly on NK cells, NKT cells, and some T cells. After CD94/NKG2A connects to its ligand HLA-E, it starts an inhibitory signal. This signal suppresses the cytotoxic and effector functions of NK cells and CD8^+^ T cells and so stops their ability to destroy target cells [9]. In many cancers, NK cells and CD8^+^ T cells that enter tumors show increased NKG2A expression. This is closely linked to immune escape. In autoimmune conditions, NKG2A acts as a shield, keeping self-reactive T cells safe from NK cell attacks [10]. During viral infections, NKG2A also helps suppress the anti-viral immune response [9]. Using monoclonal antibodies (like Monalizumab) to block NKG2A can remove this inhibition on NK cells and CD8^+^ T cells. This restores their anti-tumor cytotoxicity. Therefore, it is a promising cancer immunotherapy [11]. In contrast, NKG2C is an activating receptor. It also forms a heterodimer with CD94 (called CD94/NKG2C) and is mainly expressed on NK cells. Unlike NKG2A, when CD94/NKG2C binds to HLA-E, it sends an activating signal. This enhances NK cell to kill other cells and to release cytokines [9]. When active, NKG2C can overpower the stop signals sent by NKG2A. Together, they dynamically regulate NK cell activity [10]. In tumors, higher NKG2C expression is associated with better immune surveillance and improved prognosis [9]. NKG2C is especially important in fighting specific pathogen infections. CMV (cytomegalovirus) infection makes NK cells that have the NKG2C receptor grow more. These cells become “memory-like” adaptive NK cells, providing stronger long-term protection [10]. In HCMV (*human cytomegalovirus*) infection, NKG2C^+^ NK cells expand significantly. They show “memory-like” features: they survive for a long time, respond quickly upon reinfection, and make a lot of IFN-γ [12]. This suggests that other NK receptors might also be regulated by similar mechanisms. These mechanisms are also worth studying.

## 2. The Role of NKG2D in NK Cells

### 2.1. The Structural Characteristics and Functions of NKG2D

**Figure 1:**
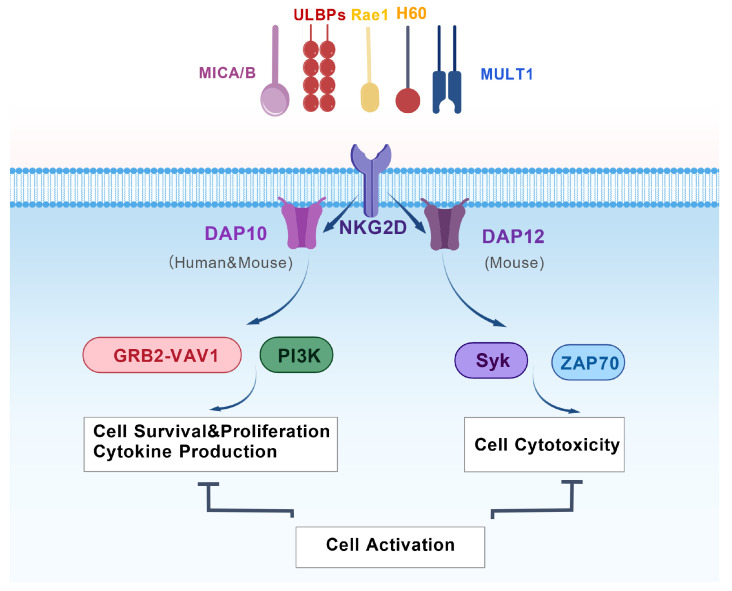
The NKG2D Receptor Complex and Species-Specific Signaling Pathways. The NKG2D activating receptor recognizes a diverse family of stress-induced ligands (e.g., MICA/B, ULBPs, RAE-1, H60, MULT1) presented on target cells. NKG2D associates with different signaling adapter proteins in a species-specific manner: it couples exclusively with DAP10 in humans but can associate with both DAP10 and DAP12 in mice. Engagement of the NKG2D-DAP10 complex recruits the GRB2-VAV1 complex and the p85 subunit of PI3K [15]. This signaling axis promotes cell survival, proliferation, and cytokine production. In mice, the NKG2D-DAP12 complex signals through the Syk and ZAP70 tyrosine kinases [15]. This pathway potently activates the cytotoxic response. The integration of signals from these distinct pathways leads to the full activation of NK cells, enabling them to eliminate stressed, infected, or transformed target cells. Created with BioGDP.com [16].

NKG2D is a type II transmembrane receptor [13] encoded by the *KLRC1* gene. The structure of NKG2D involves the extracellular domain, the transmembrane region and the intracellular region. It forms a homodimer through disulfide bonds, which is crucial for its function. The extracellular domain of NKG2D has a C-type lectin-like structure, which enables it to recognize and bind to various stress-induced ligands. Specifically, its extracellular domain consists of two β-sheet regions and two α-helices and contains four disulfide bonds that are essential for maintaining its structural stability. The transmembrane region of NKG2D is a single transmembrane structure that anchors NKG2D in the cell membrane. The presence of the transmembrane region allows NKG2D to be stably embedded in the cell membrane and to function at the cell surface. The intracellular region of NKG2D lacks signaling components. Therefore, NKG2D itself cannot directly transfer signals but requires association with specific adaptor molecules to achieve signal transduction. NKG2D has two forms: NKG2D-L (long) and NKG2D-S (short). NKG2D-L is expressed on resting mouse NK cells and on CD8^+^ T cells after they are activated. It only binds to the DAP10 adaptor protein. It provides a co-stimulatory signal, but it cannot directly activate resting cells [14]. However, NKG2D-S is only expressed when mouse NK cells are activated. It can bind to either DAP10 or DAP12. It can directly activate NK cells and enhance their killing ability [14]. Humans only express NKG2D-L, and it only binds to DAP10 (Figure 1). Therefore, resting human NK cells cannot be activated by NKG2D cross-linking alone. They need to be cultured with cytokines (like IL-15) first. After that, NKG2D alone can trigger a response [14].

NKG2D is expressed on NK cells, CD8^+^ T cells, NKT cells, γδ T cells, and on certain activated CD4^+^ T cells [17], and plays an important role in NK cell activation. NKG2D activates immune cells by recognizing ligands bound to various MHC class I molecules, such as MHC Class I Polypeptide-Related Sequence A/B (MICA, MICB) and UL16-Binding Protein (ULBP)1-6 [18] (Table 1). These ligands are usually expressed at low levels on healthy cells but are upregulated on tumor or stressed cells [19]. In NK cells, the activation of NKG2D triggers a cytotoxic response against target cells and promote the release of cytotoxic substances such as IFN-γ, TNF-α, and granzymes from NK cells, thus mediating the clearance of tumor cells. In CD8^+^ T cells, NKG2D activation requires concurrent stimulation with the T cell receptor (TCR) to enhance TCR signaling and T cell function [6]. NKG2D is involved in the recognition of tumor cells and virus-infected cells, and it plays a role in certain inflammatory diseases, autoimmune disorders, and wound-related inflammation [18]. Thus, NKG2D not only plays a key role in the immune surveillance of tumors but is also an important regulatory factor in inflammatory diseases with potential as a target for immunotherapy.

### 2.2. The Debate over Soluble NKG2D Ligands

NKG2D ligands are grouped into two categories based on their form and function: membrane-bound ligands (mNKG2DLs) and soluble ligands (sNKG2DLs). Traditionally, people believed that soluble NKG2D ligands bind to the NKG2D receptor on the cell membrane. This causes the receptor to be internalized and downregulated, for example: sMIC [23]. So, this weakens NK cells and T cells and helps tumors escape immunity [24]. However, not all soluble NKG2D ligands suppress immunity. Deng W et al. [25] found that sMULT1 causes an increase in NKG2D expression on NK cells. In the tumor microenvironment, the host’s own cells express membrane-bound NKG2D ligands. These host cells use their membrane-bound ligands to constantly and weakly stimulate the NKG2D receptors on NK cells. This leads to NK cell desensitization and NKG2D receptor downregulation. However, when the high-affinity soluble sMULT1 enters the body, it competitively binds to and occupies the NKG2D receptors on NK cells. This blocks the continuous desensitizing stimulation from the host’s membrane-bound ligands. So, it promotes tumor rejection. Serritella AV et al. [26] found that in early-stage tumors and under specific conditions, sMIC might activate immunity and delay tumor formation. The effect of sMIC on tumor formation is linked to how much stem cell markers in the tumor cells. Tumor cells that have high levels of stem cell marker expression and sMICB form tumors quickly. Those with low stem cell markers show delayed or failed tumor formation. However, after the tumor is established, sMIC switches to being immunosuppressive. It weakens NK and CD8^+^ T cell function and speeds up tumor progression. Wei L et al. [27] found that in some autoimmune diseases, large amounts of sNKG2DLs fail to cause NKG2D downregulation. This might be due to the abundant cytokine effects. Groh V et al. [28] found that in rheumatoid arthritis (RA) patient serum, TNF-α and IL-15 counteracted the downregulation of NKG2D by soluble MIC.

Therefore, the final effect of a soluble ligand (inhibition or activation) likely depends on several factors: the ligand’s own properties (such as its affinity), the presence of membrane-bound ligands that cause NK cell desensitization, the tumor’s development stage and cellular context, and the role of cytokines.

### 2.3. Function of NKG2D on NK Cells

The NKG2D/NKG2DL axis plays an important role in the immune surveillance and antitumor function of NK cells, with its activating signal being able to override inhibitory receptor signals, thereby establishing NKG2D as the “master pathway” for NK cell activation [29]. In NK cells, activation of NKG2D signaling is sufficient to trigger a direct cytotoxic response against target cells [6], a process initiated by the adaptor protein DAP10 that enhances both cytotoxic responses and cytokines production. In tumor immunotherapy, the upregulation of NKG2D ligands on tumor cells helps enhance NK cell infiltration and promote cytotoxicity against cancer cells [19], thereby playing an important role in the immune surveillance and clearance of tumor cells. In the liver, NKG2D also induces NK cells to secrete IL-17A, a cytokine that promotes liver inflammation and fibrosis [13]. Thus, NKG2D not only plays a central role in the innate immune system, but also has potential regulatory functions in adaptive immune responses [7].

### 2.4. Application Potential of NKG2D in NK Cells

In liver disease, activation of NKG2D contributes to hepatocyte proliferation following liver injury, which may promote tumor development. Increased expression of NKG2D is associated with recruitment of CD8^+^ T cells and aggravation of proinflammatory environments, which may lead to exacerbation of liver injury and increased tumor burden [18]. Moreover, activation of NKG2D involves the polarization of lymphocyte cytotoxic granules and the production of cytokines, playing a critical role in immune surveillance. In addition, the expression levels of NKG2D and its ligands hold potential as clinical targets for the diagnosis, treatment, and prognostic evaluation of cancer patients. Enhancing the expression of either the NKG2D receptor or its ligands can improve the efficacy of tumor immunotherapy. For example, possible strategies include the use of antibodies to clear soluble NKG2D ligands or employing small-molecule compounds to upregulate the expression of either the NKG2D receptor or its ligands.

The role of NKG2D in the regulation of the immune response, as well as the modulation of its ligand expression, suggests its potential value in the treatment of metabolic diseases and related complications such as atherosclerosis. In patients with acute myeloid leukaemia (AML), reduced expression of NKG2D on NK cells further highlights the importance of NKG2D in NK cell function [30]. Activation of the NKG2D axis is also considered a potential target in the treatment of metabolically associated fatty liver disease (MAFLD) and nonalcoholic steatohepatitis. Inosine pranobex (IP), as a metabolic activator, can enhance the cytotoxicity of NK cells by inducing the expression of NKG2D ligands, indicating that IP may serve as an adjuvant in immunotherapy [31]. Moreover, upregulating NKG2D ligands through the use of stress inducers such as histone deacetylase inhibitors (HDACi), heat shock, or short-chain fatty acids (SCFAs) may help treat cancers that evade immune recognition and clearance by blocking surface expression of NKG2D Ligands [19]. Therefore, NKG2D not only has potential application in tumor immunotherapy, but may also be a novel target for cancer prevention and metabolic diseases.

However, NKG2D is subject to metabolic regulation. The plasma membrane of NK cells is cholesterol-rich, and both the cholesterol content and its metabolic state can affect the distribution and function of receptors such as NKG2D on the cell membrane. The uptake and oxidation of fatty acids also play a significant role in NK cell metabolism. Certain fatty acids can influence the expression of NKG2D. For example, abnormal fatty acid metabolism may lead to an increase in lipid peroxidation products within NK cells. Metabolites produced by glycolysis and other metabolic pathways also impact the expression and function of NKG2D. These metabolic pathways provide NK cells with energy and biosynthetic precursors, which are crucial for the activation and maintenance of their effector functions. Understanding the regulatory mechanisms by which these metabolites affect NKG2D is of great importance for the development of NK cell-based immunotherapy. For instance, modulating the concentration of metabolites in the tumor microenvironment (TME) or intervening in the metabolic processes of NK cells themselves may enhance NK cell anti-tumor activity and improve the efficacy of NK cell therapies.

## 3. Metabolites Regulate NKG2D Expression as Well as NK Cell Function

### 3.1. Lipids

Lipids are an important class of heterogeneous biomolecules in organisms that perform a variety of physiological functions, including building cellular structures, maintaining homeostasis and restoring tissue function during and after inflammation. Lipids can be classified into fatty acids, glycerophospholipids (GPs), sphingolipids, sterols, triglycerides, phosphatidylinositols and their phosphorylated derivatives, cardiolipin and phosphatidylglycerol, ether lipids, and others. In addition, lipid mediators such as oxylipins, endocannabinoids (ECs), and N-acylethanolamines (NAEs) play important roles in the inflammatory response and its resolution, and are associated with various diseases including chronic obstructive pulmonary disease, asthma, Alzheimer’s disease, multiple sclerosis, obesity, and cancer [32]. The lipid metabolic network involves a number of biosynthetic nodes. Imbalances in lipid metabolism are associated with various diseases, including metabolic disorders (such as obesity, diabetes, and nonalcoholic fatty liver disease [NAFLD]), cardiovascular diseases, renal diseases, neurodegenerative disorders, and cancer [33]. Prolonged exposure to excessive lipids may lead to cellular dysfunction and death [33].

Lipids are closely linked to immune-related diseases. Specific lipid molecules, such as certain fatty acids and sphingolipids, are involved in cell signaling events that regulate the growth, development and behaviour of organisms and may also be involved in immune responses. For example, in *Caenorhabditis elegans*, specific fatty acid derivatives-such as arachidonic acid (AA) and prostaglandins (PGs)—are critical for p38 MAPK–dependent innate immune responses [34]. Under conditions of dietary restriction, ω-6 polyunsaturated fatty acids may induce autophagy and extend lifespan, a process that might be related to immune modulation and stress responses [34]. Sphingolipids play an important role in inflammation; for instance, sphingosine-1-phosphate (S1P) acts through its receptors to mediate the activation and migration of immune cells [35]. In the class of glycerophospholipids, the externalization of phosphatidylserine (PS) is associated with apoptosis, which is one mechanism by which immune cells clear damaged cells [35]. Glycerophospholipids serve as precursors for inflammatory mediators such as prostaglandins and leukotrienes (LTs). In diseases like Barth syndrome and Scott syndrome, mutations result in altered levels and distribution of phosphatidylcholine and phosphatidylserine, thereby affecting mitochondrial function [32]. Within the sterol class, cholesterol is critical for maintaining cellular membrane integrity and function, and its metabolites, such as steroid hormones, play roles in immune responses [35]. Triglycerides accumulate in metabolic diseases such as obesity and may affect immune system function. Phosphatidylinositol and its phosphorylated derivatives, such as PI(4,5)P2, play key roles in cellular signal transduction and are associated with inflammation and immune cell function [35]. Cardiolipin is involved in mitochondrial function and cell death, potentially affecting the metabolism and activity of immune cells [35]. Ether lipids participate in inflammation and immune responses, and their high levels in inflammatory cells indicate their role in immune signaling [35]. Among oxidized lipids, prostaglandins and leukotrienes play roles in asthma and other inflammatory diseases [32]. In inflammatory diseases such as myelodysplastic syndromes (MSD) and asthma, changes in the levels of endocannabinoids are related to disease severity, and alterations in the levels of N-acylethanolamines may also be associated with disease pathology [32].

Lipids are closely associated with NK cells. Metabolic products associated with dyslipidemia and hyperglycemia-such as oxidized low-density lipoprotein (oxLDL) and elevated glycation end-products (AGEs)-may promote the expression of NKG2D ligands in macrophages in vitro. Metabolism of short-chain fatty acids, in particular propionate, can induce expression of NKG2D ligands [36]. Research by Andersen et al. [37,38] found that propionate can inhibit the expression of cell surface NKG2D ligands by suppressing the process of N-linked glycosylation. Furthermore, Andersen et al. [38] discovered that metabolic activity, especially the metabolism of propionate, may enhance NK cell function by promoting the expression of NKG2D ligands (such as MICA/B). Study by Møller et al. [19] indicated that propionate metabolism can regulate the expression of NKG2D ligands MICA and MICB on tumor cells by promoting the flux of cytoplasmic citrate from mitochondria, potentially increasing the sensitivity of tumor cells to NK cell–mediated immune responses. Fatty acid metabolism also contributes to the regulation of NKG2D ligand expression in tumor cells; increased fatty acid metabolism in tumor cells may promote the expression of these ligands by supplying the lipid components necessary for their construction [19]. These findings indicate that the metabolism of short-chain fatty acids may influence the expression of NKG2D ligands through multiple mechanisms, thereby potentially enhancing NK cells’ ability to recognize and kill tumor cells. In addition, the accumulation of cholesterol in hepatic cells can induce the expression of NKG2D ligands. Once upregulated on the surface of liver cells, these ligands can be recognized by tissue-resident innate immune cells—such as γδ T cells—via their NKG2D receptors. This recognition activates the immune cells, leading to the secretion of cytokines such as IL-17A [13], which may have significant implications for the immune response in liver diseases. ApoC3 is a key component of triglyceride-rich lipoproteins (TRLs). Apoc3TG-DCs contain higher levels of intracellular lipids and, dependent on ROS, exhibit increased expression of NKG2D ligands, which in turn suppresses NK cell activity [39]. In Apoc3TG mice, elevated plasma levels of triglycerides and free fatty acids (FFAs) are observed along with impaired NK cell number and function, which is associated with lipid-induced metabolic reprogramming that leads to increased fatty acid oxidation and decreased glycolysis [39].

### 3.2. ROS

ROS are a group of highly reactive molecules in cells that play an important role in various biological processes and are closely involved in the development and progression of many diseases. Based on the site of production and function, ROS can be classified into several types: cytosolic ROS (CytoROS), mitochondrial ROS (MitoROS), peroxisomal ROS (PeroxROS) and endoplasmic reticulum ROS (Endoplasmic Reticulum ROS). Specific types of ROS include superoxide anion (O_2_^−^), hydrogen peroxide (H_2_O_2_), peroxynitrite (ONOO^−^), singlet oxygen (^1^O_2_), hydroxyl radicals (OH), as well as nitric oxide (NO) and other oxygen-containing free radicals [40,41]. They are pivotal in cellular signal transduction, metabolic regulation, immune responses, and in coping with oxidative stress [42,43]. Maintaining balanced ROS levels is crucial for preserving the physiological homeostasis of cellular organelles. An imbalance in ROS levels serves as a signal of pathological organelle stress and may lead to immune-related diseases. For instance, increased mitochondrial ROS is associated with the development of inflammatory diseases, cardiovascular diseases, diabetes, and cancer [44].

ROS play a critical role in various pathological conditions and are closely involved in the pathogenesis of atherosclerosis, diabetes, stroke, chronic granulomatous disease (CGD), autoimmune diseases and other inflammatory disorders. The link between atherosclerosis and ROS is evidenced by the impact of these molecules on endothelial cell function, vascular remodeling, and inflammatory responses, all of which drive disease progression [45]. In diabetes, hyperglycemia promotes the production of ROS, which affect vascular health through multiple pathways, including the induction of endothelial dysfunction and inflammation, thereby leading to vascular complications [45]. Following ischemic stroke, ROS are pivotal in mediating brain ischemia-reperfusion injury; they not only affect neuronal survival but may also compromise the integrity of the blood–brain barrier [45]. Chronic granulomatous disease is characterized by an inability to produce ROS effectively due to a deficiency in the NADPH oxidase NOX2, resulting in immunodeficiency and recurrent infections [40]. Furthermore, certain autoimmune diseases are associated with impaired ROS production by the NOX2 complex [40], indicating that ROS play an important role in immune regulation. ROS are an integral part of innate immunity in inflammatory diseases and infections. Although they strengthen immune defence, they can also cause tissue damage. In addition, ROS upregulate the expression of inflammatory mediators such as interleukin-1β (IL-1β), thereby contributing to the inflammatory process [41]. ROS play a role in the activation of the NLR Family Pyrin Domain Containing 3 (NLRP3) inflammasome, a key link between metabolic stress and inflammatory responses. The NLRP3 inflammasome amplifies inflammation by activating caspase-1 and promoting the secretion of IL-1β and IL-18, a process in which ROS activation is critical [42]. In addition, anti-inflammatory cytokines, such as IL-35 and IL-10, can inhibit endothelial cell activation by reducing ROS production, underscoring the important role of ROS in modulating immune responses [44]. Different T cell subsets have different sensitivities to ROS levels, which may affect their development and function, such as the involvement of TH17 cells in autoimmune and inflammatory diseases [42].

Under physiological conditions, ROS act as signaling molecules involved in a variety of biochemical reactions, including cell defence and cell cycle regulation [41]. However, when ROS formation and elimination becomes imbalanced, oxidative stress or reductive stress can occur, which is associated with a variety of diseases, including immune-related disorders [41]. Oxidative stress is linked to cell death; ROS play a role in both T cell activation-induced cell death and activation-induced cell autonomous death. Oxidative damage leads to cellular injury of DNA, proteins, and lipids, and can induce apoptosis in inflammatory bowel disease [42].

**Figure 2:**
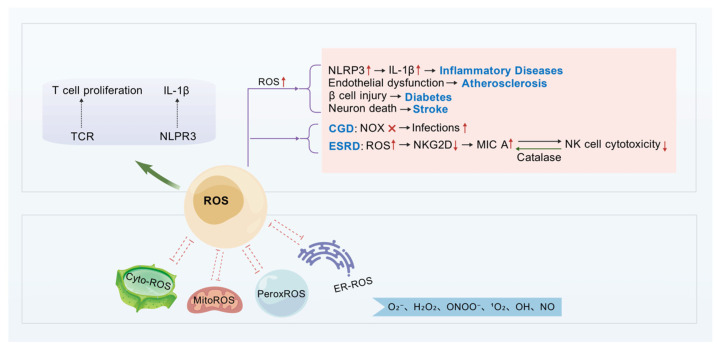
Schematic Diagram of ROS Sources, Immunological Roles, and Disease-Associated Pathways. This figure illustrates the mechanistic role of ROS in immunity and disease pathogenesis. ROS may originate from the cytosol (Cyto-ROS), mitochondria (MitoROS), peroxisomes (PeroxROS), or endoplasmic reticulum (ER-ROS), and include species such as superoxide anion (O_2_^−^). On one hand, ROS upregulate NLRP3 to promote IL-1β release, which is linked to inflammatory diseases. They also contribute to atherosclerosis via endothelial dysfunction, diabetes via β-cell damage, and stroke via neuronal death. On the other hand, dysregulated NOX activity (CGD) increases infection risk; altered ROS levels (in ESRD) impair natural killer (NK) cell cytotoxicity via the NKG2D-MIC axis, which can be modulated by catalase. Additionally, ROS participate in immune processes such as T-cell proliferation (via the TCR pathway). Created with BioGDP.com [16].

Histamine and ROS are also involved in the regulation of NKG2D expression in NK cells. Histamine upregulates NKG2D expression through interaction with the H2 receptor [30], allowing it to function even in the presence of inhibitory signals. ROS play a major role in the decreased expression of NKG2D observed in patients with end-stage renal disease (ESRD) (Figure 2). ROS can diminish NK cell cytotoxicity and reverse immunosuppression. Cancer cells with lower ROS levels are more sensitive to NK cells, whereas increasing ROS levels reduces the sensitivity of cancer cells to NK cell–mediated lysis [46]. These findings suggest that the levels and function of ROS may significantly impact NK cell activity and the sensitivity of tumor cells. During radiotherapy, the number of NK cells increases, but their function is markedly reduced, which is associated with elevated ROS levels within NK cells. The accumulation of ROS leads to excessive activation of autophagy in NK cells [47], thereby impairing their function.

### 3.3. Glucose

In the ovarian cancer microenvironment, binding of the NKG2D ligand Letal to the NKG2D receptor plays an important role in modulating T cell function. Specifically, Lethal increases the expression of the glucose transporter Glut-1 and enhances the ability of CD8^+^ T cells to uptake glucose [48]. This indicates that Letal supports T cell activation and effector function by boosting glucose metabolism. Sasawatari S et al. [49] found that T cells treated with 2-deoxy-D-glucose (2DG) exhibit enhanced antitumor activity against tumor cells, particularly those expressing NKG2D ligands. 2DG treatment upregulates the NK-specific transcription factors TOX2 and EOMES in human T cells, endowing them with NK cell–like properties, including high levels of perforin/granzymes and increased sensitivity to IL-2. By altering N-linked glycosylation, 2DG affects the glycan structures of T cell surface proteins, which may modify the interactions between these proteins and NKG2D ligands, thereby enhancing T cell–mediated anti-tumor activity. In addition, 2DG-treated T cells show upregulated surface levels of IL-2 receptors, which may increase their responsiveness to IL-2 and further strengthen the NKG2D-mediated anti-tumor immune response. Through the NKG2D–NKG2D ligand interaction, 2DG-treated T cells exhibit enhanced cytotoxicity against tumor cells, highlighting the important role of NKG2D in T cell–mediated anti-tumor immunity. Moreover, 2DG can inhibit N-linked glycosylation and reduce the expression of MICA on the surface of cells cultured under normoxic conditions [50], indicating that glucose metabolism significantly influences the N-linked glycosylation process and the expression of MICA.

In in vitro experiments, in primary cortical neurons or glial cells subjected to oxygen-glucose deprivation (OGD) treatment, increased expression of NKG2D on the surface of NK cells was induced [51]. OGD treatment mimics the hypoxia and glucose-deprived environment that occurs during cerebral ischemia, which may affect the expression of NKG2D on NK cells, indicating that hypoxia and glucose deprivation regulate NKG2D expression. A polysaccharide glucan extracted from Tornabea scutellifera was found to activate NK cells, leading to increased NKG2D expression [52]. The Tornabea scutellifera Polysaccharide Fraction 2 (TSF2) polysaccharide activates NK cells through the nuclear factor κB (NFκB) and mitogen-activated protein kinase (MAPK) signaling pathways and induces their production of various cytokines and cytotoxic molecules. This may result in elevated NKG2D expression, thereby enhancing NK cell activity.

### 3.4. Amino Acid

IL-2 increases the expression of two critical amino acid transporters in NK cells: SLC1A5 (also known as ASCT2, which is responsible for glutamine transport) and CD98 (which forms a heterodimer with SLC3A2 and is responsible for amino acid exchange). [53]. Jensen H et al. demonstrated that when the functions of SLC1A5 and CD98 are blocked using specific inhibitors, the ability of both CD56^bright^ and CD56^dim^ NK cells to produce IFN-γ and undergo degranulation in response to NKG2D stimulation is impaired, indicating that these two transporters play a key role in NK cell activation. Further studies revealed that in IL-2-pretreated NK cells, the production of IFN-γ and degranulation following NKG2D stimulation depend on the activity of SLC1A5 and CD98, underscoring that the function of these amino acid transporters is crucial for NKG2D-mediated IFN-γ production and degranulation.

Tryptophan (Trp) plays a central role in the modulation of T cell-mediated immune responses through its metabolism by indoleamine-2,3-dioxygenase (IDO). IDO activity has also been found to affect NK cell function [54]. Specifically, L-kynurenine (L-KYN), a metabolite of tryptophan produced by IDO activity, is capable of suppressing cytokine-mediated upregulation of NK cell–specific activating receptors, particularly NKp46 and NKG2D. Consequently, L-KYN-treated NK cells have a reduced ability to recognize and kill target cells via NKp46 and NKG2D.

**Figure 3:**
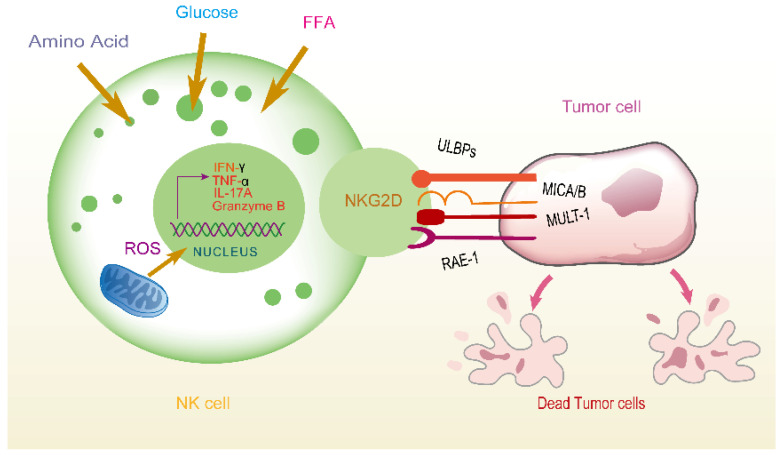
NK cells recognize tumor cells through NKG2D and induce their death. NK cells identify ligands such as ULBPs, MICA/B, and MULT-1 on the surface of tumor cells via the NKG2D receptor, triggering the activation of NK cells. Activated NK cells induce apoptosis in tumor cells by releasing factors such as IFN-γ, TNF-α, IL-17A, and granzyme B. Additionally, NK cells promote tumor cell death by producing ROS. The figure also illustrates how NK cells uptake nutrients such as glucose, FFA, and amino acids to support their functions.

Dashti Gerdabi N et al. [55] found that 5-aminolevulinic acid (5-ALA) significantly increases the expression level of the NKG2D gene in NK cells and raises the prevalence of NKG2D receptors on the surface of NK cells treated with 5-ALA. These findings suggest that 5-ALA may enhance NK cell activity by upregulating the level of NKG2D receptors. Given the important role of the NKG2D receptor in activating NK cells and killing tumor cells, 5-ALA has the potential to be used as a therapeutic approach to activate NK cells, thereby preventing cancer cell growth and metastasis (Figure 3).

## 4. The Role of NKG2D-Positive NK/T Cells in the Progression of Diseases

### 4.1. Tumor Metabolic Microenvironment

The role of NKG2D in tumor immunity is complex, as it has dual effects that can both promote and inhibit tumor growth. In some cases, NKG2D-positive NK and T cells destroy tumor cells by recognizing NKG2D ligands on the tumor cell surface [56]. However, in certain inflammation-driven cancer models, such as hepatocellular carcinoma (HCC), activation of NKG2D may actually promote tumor growth. In the context of chronic inflammation, sustained activation of NKG2D may lead to tissue damage and tumor progression, and NKG2D-positive NK and T cells may become functionally downregulated or desensitized due to continuous ligand engagement in the tumor microenvironment. These cells might also promote inflammatory responses by secreting cytokines, such as IFN-γ and TNF-α, which could further contribute to tumor growth. Despite this, NKG2D remains a potential target for immunotherapy; for example, the use of antibodies against NKG2D ligands to block their shedding may enhance the anti-tumor activity of NKG2D-positive cells.

During the immunoregulation phase, NKG2D expression has been found to define an effector CD8^+^ T-cells associated with tumor response. In CD8^+^ T cells, NKG2D signaling activates the memory-associated transcription factor Eomes, potentially through the regulation of the mTORC1 pathway, thereby contributing to memory formation. However, the distribution of NKG2D-positive cells between the tumor microenvironment and the non-tumor microenvironment may affect their function. For instance, in a Diethylnitrosamine (DEN)-induced hepatocellular carcinoma model, CD8^+^ T cells are enriched in the non-tumor microenvironment, but not within the tumor microenvironment [56].NKG2D may also have a potentially negative regulatory role in controlling TOX expression and the expression of tumor-reactive CD8^+^ T cells within the tumor microenvironment. Blocking NKG2D in Ovalbumin (OVA)-immunized mice delayed the growth of B16OVA-Luc2 tumors (a luciferase-expressing mouse melanoma cell line) and increased the presence of OVA-specific CD8^+^ T cells infiltrating the tumor [57]. Using B16OVA-Luc cells expressing OVA and luciferase allows researchers to track the OVA-specific CD8^+^ T cell response over time during tumor development. By analyzing the status of tumor antigen-specific CD8^+^ T cells at different stages, NKG2D was identified as a marker for tumor-reactive effector CD8^+^ T cells. These findings suggest that the complex role of NKG2D in tumor immunity needs further investigation to fully understand its function in different tumor types and microenvironments and to provide a basis for the development of new immunotherapeutic strategies.

The chimeric NKG2D receptor (chNKG2D) is a protein formed by the fusion of the NKG2D receptor and the CD3ζ chain, which gives T cells the ability to recognize tumor cells and mount an immune attack via NKG2D. In mouse models, it has been shown that adoptive transfer of T cells expressing chNKG2D not only significantly reduces tumor burden but also results in long-term tumor-free survival [58]. The efficacy of chNKG2D T cell therapy is demonstrated by an increase in the number of NK cells and activated host CD8^+^ T cells within the tumor microenvironment, coupled with a marked decrease in the number of Foxp3^+^ regulatory T cells at the tumor site, which play a key role in tumor-induced immune suppression. In addition, chNKG2D T cells modify the function of myeloid cells at the tumor site, shifting them from an immunosuppressive state to one that enhances T cell responses. Even when tumors are established for five weeks, the infusion of chNKG2D T cells still leads to long-term survival in mice.

Studies have shown that in transplanted tumor and genetically engineered mouse cancer models, tumor-associated macrophages can induce the expression of the NKG2D ligand RAE-1d through the action of soluble factors produced by tumor cells, in particular tumor-derived colony stimulating factor-1 (CSF-1) [59]. The complexity of the tumor microenvironment-especially the CSF-1 secreted by tumor cells-may regulate the activity of NKG2D-positive NK and T cells, with CSF-1-induced RAE-1d expression potentially enhancing these immune cells’ recognition and response to tumor cells. Thus, by targeting the CSF-1/CSF-1R axis to modulate NKG2D ligand expression, providing a potential strategy for tumor immunotherapy.

### 4.2. Diabetes

Recent studies have shown that the NKG2D receptor plays an important role in the pathogenesis of both type 1 and type 2 diabetes. During the development of type 1 diabetes, NKG2D receptor expression is increased on NK cells, CD4^+^ T cells and CD8^+^ T cells, especially auto-reactive CD8^+^ T cells, which may play a key role if NKG2D ligand expression is upregulated on pancreatic β cells. In a mouse model of transgenic diabetes (RIP-LCMV), the combined treatment of anti-NKG2D and antigen-specific regulatory T cells (Tregs) exerted a synergistic preventive effect, preventing diabetes in 75% of virus-infected mice. This suggests that blocking NKG2D during sustained pancreatitis may help preserve Treg function [60].

In the Non-Obesity Diabetic (NOD) mouse model, studies have shown that the expression of high-affinity NKG2D ligands, in particular Retinoic Acid Early Inducible-1 (RAE-1) family proteins, is increased in islet cells during the prediabetic phase. Auto-reactive CD8^+^ T cells require NKG2D for maturation, expansion and effector function, which accelerates diabetes progression. Treatment with monoclonal antibody against NKG2D can completely prevent disease progression, highlighting the critical role of NKG2D in type 1 diabetes progression [61].

Abnormal expression of the NKG2D receptor has been observed in CD4^+^ T cells in patients with type 2 diabetes, particularly in a subset of CD4^+^CD28^−^ T cells, which are more abundant compared to non-diabetic patients. In addition, these cells produce the proinflammatory cytokine IL-17, which is positively correlated with glycated haemoglobin A1c (HbA1c) levels, suggesting that they may play a greater role in patients with poor glycaemic control [62]. Studies have also found that stimulation of these cells with an anti-NKG2D monoclonal antibody induces IL-17 production, implying that NKG2D might serve as an alternative costimulatory receptor for this CD4^+^ T cell subset [62]. In addition, the frequency and absolute count of circulating NKT-like cells are significantly reduced in type 2 diabetes patients; these cells display an exhausted phenotype and diminished functionality. In these patients, NKG2D receptor expression on NKT-like cells is decreased, whereas the expression of inhibitory receptors such as Tim-3 and lymphocyte activation gene-3 (LAG-3) is upregulated. Notably, Tim-3 expression is positively correlated with HbA1c, fasting blood glucose (FBG) levels, and the duration of diabetes, suggesting that it may serve as a biomarker for the duration of the disease [63]. These findings further highlight the critical role of NKG2D in the immunopathology of diabetes and offer new perspectives for future therapeutic strategies.

### 4.3. Hepatic Steatosis

NKG2D is an activating receptor on the surface of NK cells that plays an important role in NAFLD and NASH. Research has shown [64] that NKG2D expression is significantly increased in the livers of NASH patients, the expression of NKG2D ligands such as MICA and MICB is also markedly increased in the liver of NASH patients [65]. In NASH, the increased expression of NKG2D is associated with a higher number of NK cells in the liver as observed by immunohistochemical analysis, whereas fewer NK cells are present in patients with nonalcoholic fatty liver, indicating that NK cells are activated and contribute to the progression of NASH. Activation of NKG2D enhances the ability of NK cells to kill damaged or transformed cells, and by secretion of various cytokines-including IFN-γ, IL-1β, IL-12, CCL4, CCL5 and GM-CSF-it triggers an inflammatory process via the JAK-STAT1/3 axis, leading to liver cell damage. In NK cell-deficient Nfil3^−/−^ mice, NASH formation is significantly enhanced, further confirming the facilitatory role of NKG2D in NASH progression.

Moreover, the increased rate of hepatocyte apoptosis in NASH, along with elevated expression of fibrosis markers such as α-smooth muscle actin (α-SMA) and collagen 1α mRNA, suggests that NKG2D-positive NK cells may play a role in the progression of liver fibrosis [65]. Furthermore, NKG2D activation may also contribute to the repair of liver fibrosis; in particular, the percentage of NKG2D^+^ invariant NKT (iNKT) cells is significantly increased in NAFLD patients with marked fibrosis [66]. The elevated levels of these cells correlate positively with serum glucose and BMI, suggesting that NKG2D is activated in response to liver fibrosis. In addition, NKG2D^+^ iNKT cells are positively correlated with markers of liver injury, such as aspartate aminotransferase (AST) and alkaline phosphatase (ALP), which may be biomarkers of liver fibrosis progression in NAFLD.

In NAFLD, an increase in NKG2D-positive NK cells may be associated with a stronger antiviral response. In NAFLD mice induced with a high-fat diet, infection with *murine hepatitis virus 3* (MHV-3) leads to an increase in the frequency of NKG2D-positive NK cells with a stronger antiviral activity in the early stages of infection [67]. The study results indicate that the presence of NAFLD exacerbates the severity of viral hepatitis, which may be related to the activated state of NKG2D-positive NK cells and other immune cells.

Taken together, the function of NKG2D is expressed as a marker of NK cell activation in liver steatosis-associated metabolic diseases; its increased expression is closely related to the activated state of NK cells and plays a role in immune and inflammatory responses during the development of liver steatosis.

### 4.4. Intestinal-Related Diseases

NKG2D ligands are expressed on IECs and are regulated by the gut microbiota, suggesting that NKG2D and its ligands may play an important role in gut immune surveillance. As stress-inducible surface ligands, MIC A and MIC B are normally expressed at low levels in intestinal epithelial cells and bind to the NK cell activating receptor NKG2D, forming part of the host immune surveillance against stress cells. In human Crohn’s disease, a chronic inflammatory disorder of the gut, the expression of NKG2D ligands is upregulated, which may be associated with NKG2D-mediated inflammatory and cytotoxic responses [68]. Studies have shown that certain strains of *Escherichia coli*, particularly *diffusely adherent E. coli* (DAEC), can significantly increase the cell surface expression of MIC A by interacting with CD55 (also known as decay-accelerating factor, DAF) on the cell surface [69]. This effect is mediated through the specific binding of the bacterial adhesin AfaE to its cellular receptor CD55. In addition, MIC A expression is increased in colonic biopsy samples from patients with IBD, such as Crohn’s disease, compared to controls, suggesting that MIC A expression may be involved in the pathogenesis of these diseases. The induced expression of MIC A on epithelial cells triggers the release of interferon-γ by the NKL natural killer cell line that expresses NKG2D. These findings indicate that host–bacteria interaction pathways may play a role in the pathogenesis of inflammatory bowel disease (IBD), especially in the context of bacterial triggers and genetically susceptible individuals. The MIC A-NKG2D interaction may act as a danger signal, thereby enhancing the immune response against pathogens.

Following infection of human intestinal HCT116 cells with HAdV-F41 (*human adenovirus F type*), the expression of MIC A and MIC B is significantly upregulated—particularly MIC B, which shows a marked increase in expression in infected cells. However, this does not result in a corresponding significant increase in cell surface MIC B ligands; instead, MIC B accumulates abundantly within the cell [70]. This mechanism can help virus-infected cells evade detection and destruction by natural killer cells, thereby evading immune surveillance.

IL-15 is a critical cytokine that plays an important role in the regulation of T cell function and contributes to intestinal immunity by modulating the activity of B-1a cells. B-1a cells are at the forefront of intestinal immunity as defenders, capable of producing natural antibodies (NAbs) independently of T cells. IL-15 mediates the upregulation of the activating receptor NKG2D and its adaptor protein DAP10 on B-1a cells [71] (Table 2 and Table 3). Through its interaction with B-1a cells, IL-15 activates several key signaling molecules, thereby promoting the expression of immunoglobulins that are essential for maintaining intestinal homeostasis. In organ-specific autoimmune diseases such as celiac disease, NKG2D and IL-15 lower the activation threshold of the T cell receptor and enhance the cytotoxic activity of lymphokine-activated killer cells (CTLs).

Excessive endoplasmic reticulum (ER) stress observed in intestinal epithelial cells (IEC) can trigger spontaneous intestinal inflammation. Studies have shown that ER stress specifically upregulates the regulation of NKG2D ligands, in particular mouse ULBP-like transcription 1 (MULT1) and its human homologue, in IECs through the involvement of the ER stress-associated transcription factor C/EBP homologous protein (CHOP) [99]. The increased expression of NKG2D ligands correlates with a rise in the number of NKG2D-positive innate lymphoid cells (ILCs), including NK cells and ILC1, within the intestinal epithelium. These cells accumulate during the ER stress response, and their contribution to ER stress-induced inflammation is alleviated following NKG2D blockade. Research has found that the ER stress-induced upregulation of NKG2D ligands is a key mechanism by which IECs are recognized by intestinal immune cells and subsequently drive intestinal inflammation [99]. Pharmacological blockade of NKG2D or depletion of NK1.1^+^ cells significantly improves intestinal inflammation. These findings provide a theoretical basis for potential therapeutic strategies targeting the NKG2D pathway, which may be beneficial for treating ER stress-related intestinal inflammation. Taken together, they show how ER stress in IECs promotes intestinal inflammation via the NKG2D-NKG2D ligand axis, highlighting the critical role of innate immunity in ER stress-induced intestinal pathology.

Studies have shown that infection with *Salmonella typhimurium* can increase NKG2D expression in intestinal intraepithelial lymphocytes (iIEL), while decreasing Qa-1 expression in intestinal lymphocytes [100]. Qa-1 is the ligand for the inhibitory receptor CD94/NKG2A of NKG2D. Following Salmonella infection, the number of CD8^+^ TCRγδ iIELs increases, and the expression of the activation marker CD69 on these cells is also enhanced. Notably, the expression of NKG2D on these cells is upregulated, whereas the expression of the inhibitory receptor NKG2A remains unchanged. These cells exert cytotoxicity by interacting through the NKG2D receptor with NKG2D ligands on infected cells. Blocking NKG2D recognition with an anti-NKG2D monoclonal antibody significantly reduces the cytotoxicity of iIELs against infected IECs, and in in vivo models, such blockade exacerbates *Salmonella typhimurium* infection in the gut. This indicates that NKG2D-positive cells, especially CD8^+^ TCR-positive iIELs, play an important role in mucosal immune surveillance, participating in the detection and clearance of invading pathogens. CD8^+^ TCR-positive iIELs mount an immune response against pathogens in the intestinal mucosa via NKG2D activation, thereby helping to maintain immune homeostasis in the gut.

### 4.5. Autoimmune Diseases

High blood sugar, bile acids (TDCA), gluten, and IL-15 can affect T/NK cell function by regulating NKG2D expression. The body’s metabolic state directly influences immune cell activity and the severity of autoimmune damage. NK cells play two sides in autoimmune diseases. They can suppress abnormal immune responses, but they can also promote inflammation and tissue damage. In most autoimmune diseases, NK cells help by quickly clearing pathogens to reduce immune-mediated tissue damage. Examples are systemic lupus erythematosus (SLE) and RA. In these diseases, NK cell numbers are lower, NKG2D expression is downregulated, and their function is suppressed [27]. However, in some autoimmune conditions, NK cells can become abnormally activated. When they are overactive or dysfunctional, NK cells may attack the body’s own tissues. This releases self-antigens, which can activate T cells and B cells, triggering an autoimmune response [101]. Additionally, NK cells travel to sites where autoimmune reactions occur. There, they produce a lot of pro-inflammatory cytokines like IFN-γ. This pushes the immune response toward a Th1 type, which worsens inflammation [102].

**Figure 4:**
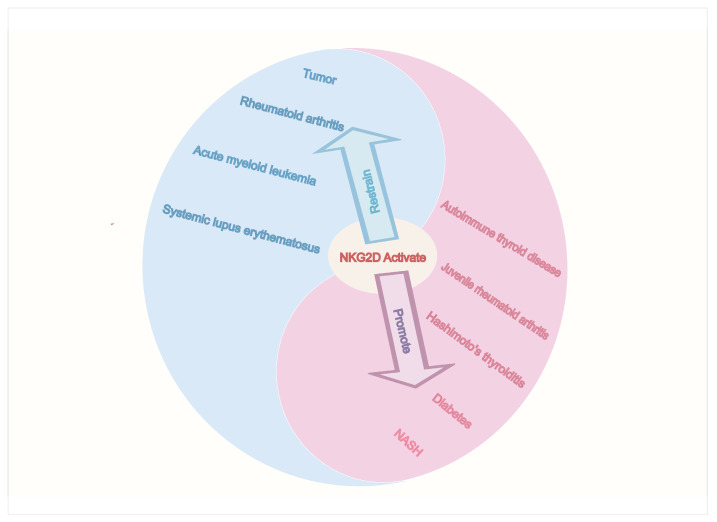
The Yin and Yang Impact of NKG2D-Positive NK cells on Diseases. NKG2D activation on NK cells plays a suppressive role in diseases such as cancer (e.g., AML) and certain autoimmune conditions (e.g., SLE, RA). Conversely, in metabolic diseases (e.g., NASH, diabetes) and other autoimmune disorders (e.g., HT, JRA, AITD), NKG2D activation promotes pathogenesis.

Wrońska K et al. [101] found that in Hashimoto’s thyroiditis (HT) patients, NK cells are involved in thyroid cell apoptosis. NK cells also worsen local inflammation by secreting pro-inflammatory cytokines like IFN-γ. Popko K et al. [102] found that in juvenile rheumatoid arthritis (JRA) patients, peripheral blood NK cells are reduced, and these cells do not work well. However, in the synovium, CD56^bright^ NK cells is increased and activated. These synovial NK cells release IFN-γ, which helps create an inflammatory microenvironment and leads to tissue destruction. In type 1 diabetes (T1D) patients, the number of peripheral blood NK cells is reduced. There are also abnormalities in their cytotoxic function and activating receptor expression. NK cells may directly destroy pancreatic islet beta cells. Alternatively, by causing cell damage, they may release self-antigens that indirectly activate autoreactive T cells. Martin TC et al. [103] identified a specific NK cell subset (CD16^+^CD56^+^CD158b^+^CD314^+^CD335^−^) in patients with autoimmune thyroid disease (AITD). This subset correlates with levels of afucosylated IgG. These cells have the activating receptor NKG2D (CD314) and a KIR receptor (CD158b), suggesting they have cytotoxic potential. In thyroid cells, two AITD-related gene variants (rs1521 and rs3094228) work by con-trolling the production of MIC-A, MIC-B, and Human Leukocyte Antigen (HLA)-C—the protein that partners with CD158b. These variants may lead to the following: making thyroid cells easier for NK cells to recognize, activating the killing pathways of NK cells, and promoting antibody-dependent cellular cytotoxicity (ADCC) against thyroid tissue (Figure 4).

## 5. The Application of NKG2D-Targeted Therapy in Cancer

### 5.1. CAR-NK Cell Therapy

Studies have shown that NKG2D CAR-T cells show promising therapeutic efficacy in the treatment of certain cancers, such as lymphoma and multiple myeloma [104]. In recent research, a novel CAR-T cell, designated 4/15NKG2D-CAR-T, was developed. This cell construct contains a chimeric antigen receptor based on NKG2D and co-expresses a designed inverted cytokine receptor (ICR). This design enhances the activity of NKG2D CAR-T cells within the pancreatic tumor microenvironment. Compared to conventional NKG2D CAR-T cells, the 4/15NKG2D-CAR-T cells exhibit increased activation, degranulation, cytokine release, and cytotoxicity against IL-4–positive pancreatic cancer cells in both in vitro and in vivo models [105]. In addition, 4/15NKG2D-CAR-T cells in vitro show higher expansion, lower depletion and a higher proportion of less differentiated T cell phenotypes. In vivo, these cells are able to destroy tumors more efficiently.

Leivas A et al. [106] designed an NKG2D-based CAR capable of recognizing multiple ligands, thereby providing broad target specificity. Activated and Expanded NK cells (NKAE) isolated from multiple myeloma (MM) patients were activated and expanded and then transduced with NKG2D-41BB-CD3ζ CAR. In mouse models, these CAR-NKAE cells effectively inhibited the growth of MM cells, with 25% of treated mice remaining disease-free. This result confirms the feasibility of NKG2D-CAR expression from autologous NKAE cells and demonstrates that these autologous CAR-NKAE cells possess enhanced anti-myeloma activity, which supports further research and development of NKG2D-CAR-NK cells for the treatment of MM.

**Figure 5:**
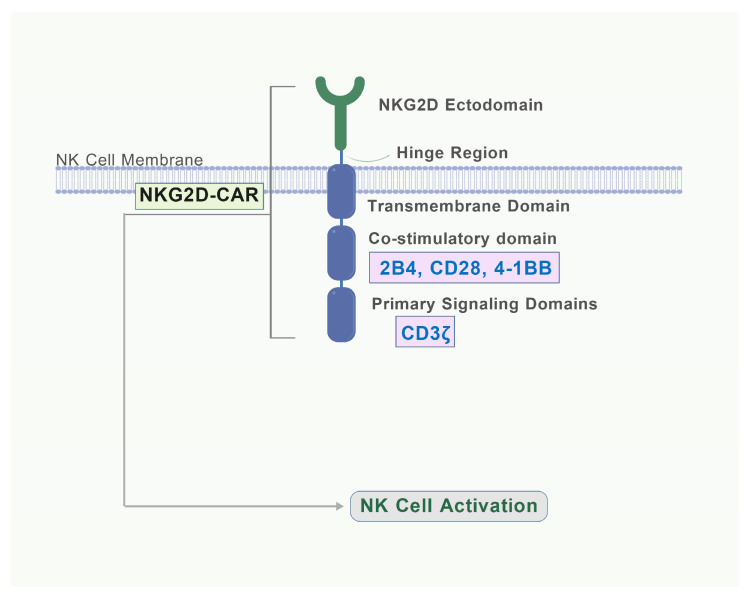
Structure of an NKG2D-based CAR for NK Cells. The extracellular domain of the CAR is derived from NKG2D, which enables the receptor to recognize a broad spectrum of stress-induced ligands on target cells. The hinge and transmembrane domains provide flexibility and anchor the CAR in the NK cell membrane. The intracellular signaling domains are composed of: A co-stimulatory domain (e.g., 2B4 [107], CD28 [108], or 4-1BB [108]) to enhance persistence and activity. The primary signaling domain CD3ζ, which contains ITAMs to initiate the activation cascade. Signal integration through these domains leads to robust NK cell activation, triggering cytotoxicity and cytokine release against target cells. Created with BioGDP.com [16].

In another study, Li Y et al. [107] focused on enhancing the tumor-killing efficacy of NK cells by designing various CAR constructs. Specifically, they developed a novel CAR, termed CAR4, which integrates the NKG2D transmembrane domain, the 2B4 costimulatory domain, and the CD3ζ signaling domain, thereby significantly enhancing the antigen-specific signal transduction of NK cells (Figure 5). By utilizing NK cells derived from human induced pluripotent stem cells (iPSC), they successfully generated cells with a typical NK cell phenotype; these iPSC-derived NK cells—particularly NK-CAR-iPSC-NK cells—demonstrated markedly enhanced antitumor activity in both in vitro and in vivo experiments after expressing CAR4. In an ovarian cancer xenograft model, NK-CAR-iPSC-NK cells significantly inhibited tumor growth and prolonged survival. In addition, these cells showed similar in vivo activity with less toxicity compared to T-CAR expressing T cells, suggesting their potential as a safer and more effective approach to cancer immunotherapy.

Li J et al. [109] developed two third-generation CAR-NK cell products, Claudin-6 (CLDN6)-CAR1 NK-92MI cells and CLDN6-CAR2 NK-92MI cells, which target CLDN6—an antigen overexpressed in ovarian cancer. In vitro experiments demonstrated that both CAR-NK cell types can specifically kill CLDN6-positive ovarian cancer cells, with CLDN6-CAR1 NK-92MI cells exhibiting stronger cytotoxicity. In mouse models, CLDN6-CAR1 NK cells effectively eradicated ovarian cancer cells from both subcutaneous and intra-abdominal sites, and when used in combination with the immune checkpoint inhibitor anti-PD-L1, they synergistically enhanced anti-tumor efficacy. These findings offer a promising new strategy for immunotherapy against ovarian cancer.

**Figure 6:**
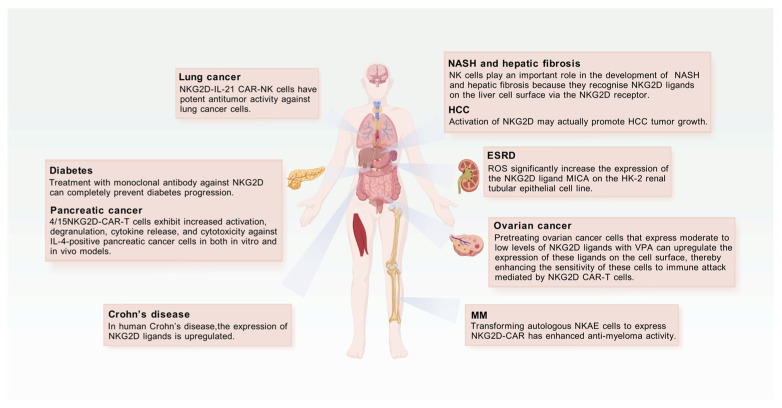
NKG2D-Mediated Immune Interventions and Mechanisms in Multiple Diseases. This figure illustrates the role of NKG2D-related mechanisms in multiple disease contexts, including lung cancer, diabetes, pancreatic cancer, Crohn’s disease, as well as NASH and hepatic fibrosis, liver cancer, end-stage renal disease, ovarian cancer, and multiple myeloma. Created with BioGDP.com [16].

To address lung cancer—a common and highly lethal malignancy—Zhang Y et al. [110] developed a novel CAR-NK cell by co-expressing NKG2D and IL-21. The expression of IL-21 was shown to enhance the cytotoxicity of NKG2D CAR-NK cells against lung cancer cells in a dose-dependent manner, thereby inhibiting tumor growth both in vitro and in vivo. In vitro, NKG2D-IL-21 CAR-NK-92 cells exhibited significantly higher cytotoxicity compared to NKG2D CAR-NK-92 cells, effectively recognizing and eliminating lung cancer cell lines. Moreover, these cells demonstrated enhanced proliferation while apoptosis and exhaustion were suppressed. In a mouse xenograft model, NKG2D-IL-21 CAR-NK-92 cells displayed effective and sustained anti-tumor activity. Compared to NKG2D CAR-NK-92 cells, mice treated with these cells showed significantly reduced tumor volumes and higher serum levels of IL-21 and IFN-γ. These results show that NKG2D-IL-21 CAR-NK cells have potent anti-tumor activity against lung cancer cells both in vitro and in vivo, offering a promising new therapeutic option for the treatment of lung cancer (Figure 6).

Additionally, Xiao L et al. [111] constructed a novel CAR structure by fusing the extracellular domain of NKG2D with DAP12. The NKG2D RNA CAR significantly enhanced the cytotoxicity of NK cells against various solid tumor cell lines and demonstrated notable therapeutic efficacy in in vitro experiments. In mouse models, NK cells modified with the NKG2D RNA CAR exhibited significant therapeutic effects against established solid tumors, and repeated injections of these CAR-NK cells effectively delayed tumor progression. Treatment of three patients with metastatic colorectal cancer with local injection of CAR-NK cells reduced ascites formation in two patients and significantly reduced the number of tumor cells in the ascites; in a third patient, liver metastases achieved a full metabolic response confirmed by PET-CT after treatment. These results support the potential of NKG2D CAR-NK cells as a promising new option for cancer immunotherapy.

### 5.2. Vaccine

Badrinath S et al. [112] developed an innovative cancer vaccine specifically targeting the stress proteins MIC A and MIC B (MIC A/B), which are expressed in various human cancers due to DNA damage, while their expression levels in normal cells are typically low or nearly undetectable. This vaccine works by inducing the host to produce antibodies that effectively increase the density of MIC A/B proteins on the surface of tumor cells, thereby not only enhancing dendritic cell antigen presentation but also significantly boosting the cytotoxic function of NK cells. The vaccine has demonstrated outstanding anti-tumor efficacy, effectively controlling the growth of subcutaneous B16F10 and EL4 tumors, and even inhibiting tumor metastasis following surgical resection of primary tumors in mouse models. Notably, the vaccine exhibits significant therapeutic effects against tumors lacking MHC class I molecules through the synergistic activation of NK cells and CD4^+^ T cells. In studies using non-human primates (macaques), the vaccine showed good safety and immunogenicity, with all vaccinated macaques generating antibodies against MIC A and MIC B. In addition, the vaccine effectively recruits and activates various T and NK cells in the tumor microenvironment, thereby providing protective immunity against drug-resistant tumors with normal immune evasion mechanisms.

Torres N et al. [113] created a Brucella Lumazine Synthase (BLS)-MICA fusion protein vaccine. They combined the human MICA extracellular region with Brucella BLS protein to make it more immunogenic. They immunized mice with this vaccine, and it induced the production of anti-MICA polyclonal antibodies. In a prevention model, immunized mice showed a clear delay in tumor growth after being injected with MICA-positive tumor cells. Next, they took serum containing anti-MICA polyclonal antibodies from immunized mice and injected it directly into mice that already had tumors. This significantly slowed the growth of two types of MICA-positive tumors: EL4 lymphoma and MB49 bladder cancer. Tumor cells shed MICA and other NKG2D ligands through proteolysis to escape immune surveillance. The released soluble MICA then binds to NKG2D and causes it to be downregulated and degraded. The binding of MICA on tumor cells to NKG2D on NK cells is a key step in triggering ADCC by NK cells. The therapeutic effects of BLS-MICA immunization included clearing sMICA and triggering anti-MICA antibody-mediated ADCC. It also helped shift tumor-associated macrophages toward the M1/pro-inflammatory type and brought more antigen-experienced CD8^+^ T cells into the tumor. Torres N et al. [113] shows that antibodies targeting MICA can block this shedding, so the NKG2D-mediated killing of tumor cells becomes stronger. This strategy can be used not only for treatment, but also as a postoperative supplement or a preventive measure.

Survivin is an inhibitor of cell death proteins. Too much Survivin in tumors is linked to lower patient survival, more cancer coming back, and resistance to treatment. In both preclinical and clinical trials, Survivin-based DNA vaccines have been shown to cause T cell-mediated anti-tumor responses, and they did not cause serious toxicity. The NKG2D ligand H60 improved the anti-tumor effect of a DNA vaccine that encodes mouse Survivin [114]. When the NKG2D ligand H60 and Survivin are expressed together in the same vaccine, the vaccine can activate dendritic cells, NK cells, and CD8^+^ T cells. It also reduces negative regulation by CD4^+^CD25^+^ regulatory T cells and can induce long-term T cell memory [115]. This shows that the vaccine activates both innate and adaptive anti-tumor immunity, and therefore it better prevents tumors from different origins and with different NKG2D expression levels.

## 6. Conclusions

After a thorough investigation of the critical role of NK cells and their surface receptor NKG2D in the immunological surveillance of tumors and metabolic diseases, we conclude that the NKG2D/NKG2D ligand axis is key in the regulation of the immune response and in the maintenance of overall health. NKG2D not only plays an important role in innate immunity but also has potential regulatory functions in adaptive immune responses. This discovery provides an important theoretical basis and therapeutic target for the development of new immunotherapeutic strategies. As the NKG2D signaling pathway continues to be investigated, we hope to elucidate its complex mechanisms in various diseases, thereby offering new perspectives for the treatment of tumors, metabolic disorders and inflammatory diseases. Therapies targeting NKG2D, including CAR-NK cell therapy and vaccine development, have already shown potential in boosting anti-tumor immunity and improving metabolic disease outcomes. Further optimisation and clinical application of these strategies could lead to more effective and personalized treatment options for patients.

In the field of cancer immunotherapy, NKG2D-targeted strategies have demonstrated tremendous potential. By enhancing the ability of NK cells to recognize and kill tumor cells, activation of NKG2D may serve as an effective therapeutic approach. Moreover, NK cells engineered with CAR technology have shown potent cytotoxicity against tumor cells in both in vitro and in vivo studies, providing a novel strategy for cancer treatment. This approach is particularly promising for malignancies such as pancreatic and lung cancers, where conventional treatments often yield limited results. In metabolic diseases—especially NASH and type 2 diabetes—the role of NKG2D is equally noteworthy. Activation of NKG2D may exacerbate liver inflammation and fibrosis, and in type 2 diabetes, NKG2D-positive T cells may play a greater role in patients with poor glycemic control. These findings suggest that NKG2D could be a new therapeutic target for treating these metabolic disorders. In gut-related diseases, the expression of NKG2D and its ligands is regulated by the gut microbiota, indicating that NKG2D may play an important role in intestinal immune surveillance. In inflammatory bowel disease, the upregulation of NKG2D ligands may be linked to NKG2D-mediated inflammatory and cytotoxic responses, providing a theoretical basis for potential therapeutic strategies targeting the NKG2D pathway, which could be beneficial in treating intestinal inflammation. In the area of vaccine research, strategies targeting NKG2D ligands have shown the potential to activate both the innate and adaptive immune systems, offering new approaches to overcome tumor-induced peripheral tolerance. By activating the NKG2D signaling pathway through vaccination, it is possible to enhance the anti-tumor activities of NK cells and T cells, thereby providing a novel perspective for cancer immunotherapy.

In conclusion, NKG2D plays an important role in the pathogenesis of various diseases and its use in immunotherapy is very promising. Future research should focus on elucidating the complex regulatory mechanisms of the NKG2D signaling pathway and how modulating this pathway can enhance immune surveillance and treat related diseases. In addition, the safety and efficacy of NKG2D-targeted therapeutic strategies in clinical applications need to be further investigated in order to provide cancer patients with more effective treatment options. A deeper understanding of changes in NKG2D expression and function in different disease conditions will facilitate the development of more accurate diagnostic tools and therapies. In addition, NKG2D studies in metabolic diseases offer new opportunities for the development of innovative therapeutic strategies. With continued advances in immunology and molecular biology techniques, as well as a better understanding of the NKG2D signaling pathway and the ongoing development of new immunotherapeutic strategies, there is reason to believe that NKG2D research will bring revolutionary changes to human health and that therapies targeting NKG2D are likely to become an important tool in the treatment of cancer and metabolic diseases.

## Figures and Tables

**Table 1:** Summary of NKG2D Ligands and Their Functions in Humans and Mice.

Name	Source	Function	References
MICA/MICB	Human	It makes the killing effect of NK cells stronger by acting on the NKG2D receptor.	Okita R et al., 2019 [20]
ULBP1-6	Human		
Rae1	Mouse	It stimulates tumor immunity.	Diefenbach A et al., 2001 [21]
H60	Mouse		
MULT1	Mouse	Soluble MULT1 greatly enhances the cytotoxic function of CD8^+^ T cells through the NKG2D receptor.	Legroux L et al., 2019 [22]

**Table 2:** Regulation of NKG2D and Its Ligands by Various Cytokines.

Name	Function	References
IL-2	Upregulation of NKG2D expression	Verneris MR et al., 2004 [72]
IL-4	Downregulation of NKG2D expression	Marçais A et al., 2013 [73]
IL-10	Downregulation of MICA expression	Serrano AE et al., 2011 [74]
IL-12	Upregulation of NKG2D expression	Dean JW et al., 2020 [75]
IL-15	Upregulation of NKG2D expression	Ghosh AK et al., 2017 [71]
IL-18	Upregulation of NKG2D expression	Song H et al., 2006 [76]
IL-21	Downregulation of NKG2D expression	Burgess SJ et al., 2006 [77]
TNF-α	Upregulation of NKG2D expression	Groh V et al., 2003 [28]
TGF-β	Downregulation of NKG2D expression	Song H et al., 2006 [76]
IFN-α	Upregulation of MICA expression	Zhang C et al., 2008 [78]
IFN-β	Upregulation of NKG2D expression	Tahrali I et al., 2019 [79]
IFN-γ	Downregulation of MICA expression	Zhang C et al., 2008 [78]
CSF-1	Upregulation of RAE-1δ expression	Thompson TW et al., 2018 [59]
TL1A	Upregulation of NKG2D expression	Tougaard P et al., 2015 [80]

**Table 3:** Therapeutic Strategies Targeting NKG2D.

Name	CAS Number	Function	References
Entinostat	209783-80-2	A certain HDAC inhibitor can upregulate the expression of MIC A and MIC B, thereby enhancing NK cell cytotoxicity.	Zhu S et al., 2015 [81]
Romidepsin	128517-07-7	An HDAC inhibitor can enhance NK cell cytotoxicity.	Satwani P et al., 2014 [82]; Chu Y et al., 2017 [83]
Temozolomide	85622-93-1	It can upregulate the expression of MIC A, MIC B, ULBP2, RAE-1, and MULT-1.	Weiss T et al., 2018 [84]
Cisplatin	15663-27-1	Upregulation of MIC A and MIC B expression enhances NK cell cytotoxicity.	Okita R et al., 2016 [85] [85]
MG132	133407-82-6	Upregulation of MIC B expression enhances NK cell cytotoxicity.	Luo D et al., 2019 [86]
Bortezomib	179324-69-7	Upregulation of MIC B and ULBP1 expression enhances NK cell cytotoxicity.	Lee YS et al., 2018 [87]
MMP inhibitor		For example, MMPI-IV can upregulate the expression of MIC A, MIC B, ULBP2, and ULBP3, thereby enhancing NK cell cytotoxicity.	Le Maux Chansac B et al., 2008 [88]
7C6 monoclonal antibody		Upregulation of MIC A and MIC B expression enhances NK cell cytotoxicity.	Ferrari de Andrade L et al., 2018 [89]
Hematoporphyrin		Upregulation of MIC B, ULBP1, ULBP2, and ULBP3 expression enhances NK cell cytotoxicity.	Park MJ et al., 2011 [90]
Cytokine		Cytokines such as IL-2, IL-12, IL-18, and IL-15 can upregulate the expression of the NKG2D receptor, thereby enhancing NK cell cytotoxicity.	Song H et al., 2006 [76]; Konjević G et al., 2010 [91]; Ghasemi R et al., 2016 [92]
CH_3_SeH		It can upregulate the expression of MIC A and MIC B, thereby enhancing NK cell cytotoxicity.	Hagemann-Jensen M et al., 2014 [93]
miR-20a		Downregulation of MIC A and MIC B expression reduces NK cell cytotoxicity.	Xie J et al., [94]., 2014; Shen J et al., 2017 [95]
EGFR activator		It can upregulate the expression of MIC A and ULBP2, thereby enhancing NK cell cytotoxicity.	Vantourout P et al., 2014 [96]
miR-34a		It can both induce and reduce MIC B expression, thereby enhancing NK cell release of the cytotoxic effector IFN-γ.	Zhou MT et al., 2018 [97]
Bufalin	465-21-4	It can directly balance the stimulatory and inhibitory receptors on the surface of NK cells and indirectly activate NK cells by inhibiting MIC A shedding, thus preventing immune evasion and enhancing NKG2D-dependent immune surveillance.	Fu R et al., 2021 [98]

## Data Availability

No new data were created or analyzed in this study. Data sharing is not applicable to this article.

## Abbreviations

Abbreviation	Full Name
2DG	2-deoxy-D-glucose
5-ALA	5-aminolevulinic acid
AA	Arachidonic Acid
ADCC	Antibody-Dependent Cellular Cytotoxicity
AITD	Autoimmune Thyroid Disease
AML	Acute Myeloid Leukaemia
BLS	Brucella Lumazine Synthase
CAR	Chimeric Antigen Receptor
CGD	Chronic Granomatous Disease
CLDN6	Claudin-6
CMV	Cytomegalovirus
CSF-1	Colony Stimulating Factor-1
DAEC	Diffusely Adherent E. coli
ECs	Endocannabinoids
ESRD	End-Stage Renal Disease
HAdV-F	Human Adenovirus F type
HDACi	Histone Deacetylase Inhibitor
HT	Hashimoto’s Thyroiditis
IBD	Inflammatory Bowel Disease
IDO	Indoleamine-2,3-dioxygenase
IP	Inosine Pranobex
JRA	Juvenile Rheumatoid Arthritis
L-KYN	L-kynurenine
MHV	Murine Hepatitis Virus
MM	Multiple Myeloma
MULT	Mouse ULBP-like Transcript
NKAE	Activated and Expanded NK cells
NLRP3	NLR Family Pyrin Domain Containing 3
NOD	Non-Obesity Diabetic (mouse)
OGD	Oxygen-Glucose Deprivation
RA	Rheumatoid Arthritis
RIP-LCMV	Rat Insulin Promoter-Lymphocytic Choriomeningitis Virus
SLE	Systemic Lupus Erythematosus
TDCA	Taurodeoxycholic Acid
TOX	Thymocyte Selection-Associated High Mobility Group Box
TSF2	Tornabea scutellifera Polysaccharide Fraction 2
ULBP	UL16-Binding Protein
